# Book Reviews

**Published:** 1819-02

**Authors:** 


### CRITICAL ANALYSIS
### OF RECENT PUBLICATIONS,
IN THE
### DIFFERENT BRANCHES OF MEDICINE, SURGERY, &c.

*Medico-Chirurgical Transactions, published by the Medical and Chirurgical Society of London.* Vol. IX. Part I.—Longman and Co. 1818.

*(Continued from p. 91.)*

II.—*History of a Case of Cæsarean Operation, in which the Lives of the Mother and Child were both preserved.* By J. J. Locher, M.D. Town Physician of Zurich. Communicated by J. A. Albers, M.D. of Bremen.

IT is extraordinary that the operation which is here reported to have been performed with perfect success, should, almost without exception, have been attended with an opposite result in this country. The records of continental surgery abound with instances of the favourable termination of the Cæsarean section; whilst the fatal issue of those cases in which it has been adopted with us, has for many years proscribed almost the bare mention of it. To what circumstances are we to ascribe a difference so marked in the success of an operation not difficult in the practice? Perhaps the practitioners of the continent, having less dread of the consequences,—the impracticability of natural delivery being determined,—have recourse to the operation in an early stage of labour, whilst the constitution is still tranquil and its powers unimpaired. If this moment be allowed to pass, and we wait until a protracted series of ineffectual efforts have worn out the patient, and brought the parts to be operated upon into a state of high irritation, if not of incipient inflammation, we lessen surely, to a fearful extent, the chances for our patient's life. As cases do unfortunately occur, where, from mal-conformation, the natural mode of delivery is physically impracticable, and the lives of both mother and child will necessarily, but for some interference of art, be sacrificed; it becomes unquestionably the duty of the practitioner, under such circumstances, to suggest and to press the adoption of a measure, which has the success of repeated experience for its sanction: and this at an early period of labour, or the chance of success will not remain.

In the case here related, Dr. Locher performed the operation, within the first twenty-four hours, by an incision in the linea alba, extending from immediately below the navel for eight or ten inches. After the parietes of the abdomen were divided, and the uterus brought into view, an uneven portion of the surface of this viscus was selected, in order to avoid the placenta, and an incision sufficient to admit the index of the left hand was made, from which, the finger serving as a director, the aperture was dilated with the knife from six to eight inches. The child with its membranes now presented, but without any water. The hæmorrhage till then was but very trifling. The child being liberated from the uterus, respiration immediately commenced, and the funis was divided. The placenta was found on the right side of the womb, lying almost free, and was removed. At this moment a violent bleeding arose from the bottom of the uterus. The blood was quickly absorbed by a sponge, to leave the organ to its own contraction, and to close the integuments. Dr. Locher observes, that he followed this method from having observed, two years previously, in a case where the woman died eight days subsequently to the operation, that the uterus was quite contracted, and the labia of the wound in it almost entirely united. The external teguments were now brought together by five sutures, lint and adhesive straps applied, and the whole confined with a couple of compresses and a broad bandage. The woman was put to bed; and neither fainting nor any other accident befel her. She took some broth; an opiate was given, and tranquillity enjoined. The lochia flowed *per vaginam*. Every thing went on well, with the exception of a sudden and somewhat alarming disturbance on the fourth day, from some undetected cause. This passed off, and the patient progressively improved. The ligatures came away towards the end of a fortnight, and the wound healed favourably. The menses re-appeared, somewhat irregularly, in the seventh week, and afterwards observed their due course. In the eighth week the woman transacted most of her domestic concerns, and was no more confined to her bed during the day. In the twelfth, she paid a visit to the Doctor, in the best health, accompanied by her infant.

A small spot, not exceeding two or three lines in length and breadth, in the middle of the wound, continues open, in spite of all remedies; and, when thought to be healed, shortly re-opens; but without detriment to the patient.

III.—*A Case of Inguinal Aneurism cured after the use of Compression.* By J. A. Albers, M.D. of Bremen.

In this case compression was made on the aneurism by an instrument constructed on the principle of a tourniquet, whereby regulated pressure could be made. After two months' constant application, so much pain in the part and œdema of the limb were produced, that it was relinquished. The tumor now attained a considerable size, being as large as a goose's egg, red and inflamed, and pulsated with violence. The whole thigh was extremely painful, and cold. Frictions with flannel were employed, and a low diet and antiphlogistic treatment; but no loss of blood. After the patient had remained a week quietly in bed, the pain and pulsation abated; the compress was again applied, without much inconvenience. The size of the aneurism now diminished, the pain and swelling of the thigh subsided, and the patient was soon able to walk with the help of a stick. The amendment was uninterruptedly progressive for six or seven months; after which time no further pulsation could be distinguished in the inguinal region, and the swelling and pain had wholly disappeared: the compress was no longer used. Some months afterwards, the thigh remained rather thin, with some trifling œdema, and a little weariness after much walking was experienced; but, in other respects, the man was well enough to resume his employment as a sailor.

Dr. Albers does not adduce this treatment in preference to the operation, but as an instance of success, which may give a ground for reasonable hope in cases where we are restricted from the use of more prompt and more certain means of relief.

IV.—*Case of Cynanche Laryngea;* by Dr. Arnold, of Stamford. Communicated by Dr. Baillie.

This is a well-marked instance of the disease yielding to a judicious and active treatment, by general and local abstraction of blood, and a free exhibition of submuriate of mercury; assisted by enemata and some other auxiliaries.

V.—*Some Observations on the Cure of Hydrocele of the Tunica Vaginalis Testis, without procuring an Obliteration of the Sac;* by Kinder Wood, Esq. Member of the Royal College of Surgeons, and Surgeon in Oldham.

Mr. Wood, in this paper, relates some cases followed by a success so marked as to claim for his treatment, in our opinion, the deliberate consideration of the profession. The method which has hitherto been employed for what is termed the radical cure of hydrocele, certainly does not leave the parts in a state so nearly natural as the means here recommended; and, if a more enlarged use establish the same results, we must consider the practice of this part of surgery importantly improved. Mr. Wood's treatment appears, as far as we can judge from his cases, to restore the lost balance of action betwixt the exhalant and absorbent systems of the part, without procuring, as in the ordinary mode, an abolition of the secreting surfaces. This operation is a slight extension of the palliative one performed simply for the evacuation of the fluid. He makes a puncture with a broad-shouldered lancet, which, from its form, produces a more extensive incision in the outer integuments than in the tunica vaginalis. After the escape of the fluid, a small portion of the membrane presents at the orifice: this he engages with a small dissecting hook, and, bringing it forward, removes it with a pair of scissors. The wound is closed with adhesive plaster, the parts supported, and quietude enjoined. In the cases here recorded, inflammation appears to have been induced; but, from the accompanying symptoms, of moderate character, and not followed by an adhesion of the two membranes. This results, no doubt, from there not being two inflamed surfaces in opposition, as is the case when an irritating fluid is thrown into the cavity, as in the ordinary treatment. The local inconvenience and general disturbance supervening on the operation were so slight as to leave the patients very shortly in a state of entire exemption from suffering of every kind connected with the particular affection. One instance is recited, wherein Mr. Wood operated, against his judgment, on a patient labouring under pulmonic disease; and here the symptoms ran somewhat high, but less formidable, he thinks, than might have been anticipated from any other plan of operation, and the termination was very favourable. It is worthy of remark, too, that this patient, with the subsidence of the symptoms incurred by the operation, was relieved from the previously-existing disease of the chest.

VI.—*Case of Hereditary Icthyosis;* by P. J. Martin, Esq. of Pulborough, in Sussex. Communicated by Mr. Cline.

This cutaneous affection, occurring in a mother and child, forms the subject of two well-executed plates, that afford a good idea of the appearance of the morbid surface. This condition began to manifest itself in both subjects when about three months old, and is extended nearly over the whole skin, except that of the head and neck. It began without any marked constitutional derangement, and exists independently of observable disease of the system. But, as Mr. Martin remarks, these individual cases are more curious from a display of the hereditary tendency of the affection, than useful as affording any practical deduction. They hold at present an unprofitable, though not an unmerited, place in the records of anomalous aberrations from healthy structure.

VII.—*Experiments on the Transfusion of Blood by the Syringe;* by JAMES BLUNDELL, M.D. Lecturer on Physiology at Guy's Hospital. Communicated by Mr. CLINE.

Dr. Blundell has been led to institute experiments on the disused practice of transfusion of blood, from witnessing the death of a woman from uterine hæmorrhage; the results of which are interesting in a physiological point of view, and we think in some instances may contribute to the preservation of human life. The facts developed in this enquiry are—that blood, exposed for a short time in an open vessel, and then transmitted by means of a syringe into the veins of an animal exhausted by hæmorrhage, is still fitted for the purpose of resuscitation and of maintaining vital action. The time which blood may be allowed to stagnate in the cup, without destroying its power of restoring vitality, seems to vary with the difference of coagulating tendency in the blood of different animals. Arterial blood of the same animal may be transfused into the veins by the syringe, continuing the operation so long as to bring the whole quantity of circulating blood several times through the apparatus; and this without apparent detriment. It seems probable, from these experiments, and others alluded to, performed by Mr. Goodridge and Dr. Leacock, of Barbadoes, that the blood of one species of animal cannot be safely substituted for that of another; although it appears that this exchange has been made with impunity. Venous blood has the same power of restoring life as arterial. Air (atmospherical and from the lungs), water, and even weak wine, were injected in these experiments on dogs, in small quantities, without serious inconvenience to the subject. The animals operated on have been suffered to lie for some seconds in a state of apparent death before injection was commenced, and have been immediately restored. From all these circumstances, Dr. Blundell infers that we might, with every prospect of advantage and safety, transfuse, by means of the syringe, human venous blood into the veins of a patient dying by any sudden evacuation of this vital fluid. In cases of such desperate character, and where the means could be promptly commanded, we should certainly consider it a duty to resort to the experiment. The apparatus used by Dr. Blundell is ingeniously adapted to the purpose; but an intelligible description of it could not be conveyed without a graphical representation.

VIII.—*History of the Progress and Enquiry into the Causes of the Yellow Fever, as it appeared in the Island of Antigua in the Year* 1816; by A. MUSGRAVE, M.D. of Antigua. Communicated by Dr. FERGUSON, Inspector of Hospitals.

For some time preceding the month of June, 1816, Dr. Musgrave says that the island of Antigua was as exempt from disease as one of equal population could well be expected to be under any circumstances, or in any variety of climate. A few sporadic cases of fever occurred at intervals, but not fatal, and in general they readily yielded to the treatment adopted. These cases appear to have been mostly resulting from the operation of ordinary exciting causes of fever,—exposure to alternations of temperature; which in a tropical climate are more intense, and commonly applied to subjects powerfully predisposed to yield to their influence.

On the 18th of June, 1816, two seamen were admitted into the parochial Hospital, situated on Rat-Island, labouring under symptoms of fever. One had but just become affected with the disease, the other was on the close of the third day. Both these patients, from the state of their symptoms at this time, failed to impress Dr. Musgrave, less familiar with the fever of the climate, with apprehensions as to the nature of their malady: his partner, Dr. Daniel, however, soon after recognized in them circumstances of an alarming character, which led him to form an unfavourable prognosis respecting it. They both died on the fifth day, after manifesting all the symptoms which are usually marked as characteristic of yellow fever.

These cases appear to have been the first decided instances of the ensuing prevalent epidemic: it therefore becomes important, for the adjustment of some still discordant opinions upon the nature of the disease, to ascertain, with all possible accuracy, the peculiar circumstances under which these two individuals had acquired the malady. These two seamen arrived in a ship, from Charlestown, on the 25th of April, 1816; at which time the crew was perfectly healthy. They were discharged on the 5th of May following. On being received into the hospital, they were reported to be out of employ, and to have been taken ill at the house of a woman of colour, in the north-west part of the town called the Point, where they had boarded for some weeks. Succeeding instances of the disease occurred, originating in the same quarter of the town; and for some time they were confined to it. They successively spread over other parts of the town and vicinage: with the localities of this first nidus of the distemper, it is interesting, therefore, to become acquainted.

"The houses there are not merely exposed to currents of air which have previously traversed a marshy surface, but they are absolutely standing in a swamp. Three or four streets cannot with safety be passed on horseback; and the path which those on foot are obliged to select with the utmost circumspection, is afforded only by artificial ground. The house of the coloured woman, from which the two first cases emanated on the 18th of June, forms one of a row terminating the town to the north-west. From the very threshold of her door, stretching to the northward and north-west, an extent of marshy ground proceeds for nearly two miles, and also to the north-east for a considerable, though not so great a distance: and, as I have already stated that three or four streets to the southward of this are absolutely built in a swamp, the inference is plain, that, from whatever quarter the wind may blow, it will bring with it a noxious impregnation; nay, from the imperfect manner in which these huts are constructed, miasmata must spring up from beneath the very beds they contain. When we reflect on these indisputable facts, we are constrained to wonder, not that an epidemic should have originated there in 1816, but that every revolving year does not bring with it melancholy examples of the baneful nature of the exhalations which we should suppose are necessarily generated in such a situation. In fact, although nothing so extensive is to be met with elsewhere, every part of St. John's may be deemed (with few exceptions) more or less exposed to the effects of marsh effluvia. Swampy tracts are seen just beyond the southern outskirts of the town, and to the eastward they are abundant at some little distance, although a good deal intercepted by what is called Government-hill."

These facts surely speak very pointedly as to the origin of the disease, which we cannot hesitate to consider as endemial. As a proof, too, that the district abounded at this season with the noxious principles universally incident to similar localities, the natives and those whose constitutions, by long residence, were assimilated to the climate, were more than ordinarily obnoxious to attacks of intermittent fever. One cause, therefore, of febrile disease was here manifestly in full activity; but it encountered, in the different classes of its victims, habits and predispositions of obviously different characters, and its effects seem to have experienced an uniformly correspondent modification. The testimony of all the facts comprised in Dr. Musgrave's communication is decidedly adverse to the suspicion that the epidemic was of foreign origin: this, therefore, forces upon us a reiterated conviction, that in the peculiarities of the district we are to search for the sources of the disease. Conformable to the experience of other observers, in this instance, those recently arrived from another climate, those who had not been long settled here, or even strangers from contiguous islands, were most susceptible of the influences exciting yellow fever: the native inhabitants were exempt. When the disease had apparently suspended its course, if an European chanced to arrive, even from another island, he was usually speedily attacked.

With respect to the contagious nature of yellow fever, Dr. Musgrave is very decidedly a disbeliever; and he is maintained in this opinion, he says, by all the practitioners of longer experience in the disease than himself, with whom he has conversed.

The evidences adduced on this point are strong; amongst them we should be disposed to rank the following as conclusive.

"His Majesty's ship Brazen being detained in our harbour on some duty, her crew became dreadfully unhealthy; and, at length the surgeon himself being confined, in November it became necessary to land the sick. The naval hospital at English Harbour having been closed on breaking up the establishment there, an order was given for their admission into the military hospital at St. John's; and thirteen or fourteen cases were accordingly placed under my care, as acting medical officer at this port. They proved as malignant in their progress as cases could possibly be. Several privates of the 4th West-India regiment were in a contiguous ward,* and in so small an establishment I could not have prevented, had I been most anxious to do so, the freest communication between them and the seamen. The bedding, &c. covered with black vomit and hæmorrhages from those who died with these fatal symptoms, (although every precaution was taken to ensure cleanliness,) were not destroyed; and yet, neither during the time the patients continued there, nor after the hospital was cleared of them (now upwards of three months), has one individual, who was in attendance, or in any manner connected with the detachment at the barrack, laboured under a disease at all resembling yellow fever. Indeed, from what I saw in that and other instances, I feel convinced that the disease cannot be propagated even by sub-

* The wards of that hospital had no doors; the communication, therefore, was perfectly open, and the intercourse unrestrained.

jects brought from on ship-board, whence the most plausible arguments are derived in favour of contagion."

— "Nay, I once saw the wife of a sailor stowed into the same cot in which her husband lay with this fever in its worst form, and yet escape, under every predisposition that could be conceived in a full-blooded and unassimilated subject.

These instances bear strongly on the point, and others in confirmation are likewise adduced, which appear to prove the noncontagious nature of the original disease; but it is justly remarked, the undue accumulation of human effluvia in a crowded ship or prison will, in the West-Indies as well as in other climates, prove productive of temporary pestilence; but this cannot be allowed as an evidence of the contagious character of the disease in question. During the course of the epidemic of Antigua, it was demonstrated that one attack of this fever has no preservative power against subsequent ones. Several instances of a second occurrence of the fever in the same individual, and that frequently fatal, came under the immediate cognizance of Dr. Musgrave in the space of a few months.

The diagnosis of this disease, in the earlier stages or in its milder forms, is indistinct, and, where it assumes a more decided character, is commonly useless; as the symptoms which are usually considered as distinctive of the fever rarely manifest themselves but to the extinction of hope. The black vomit is not an invariable attendant. Dr. Musgrave remarks, that, when the stomach is the organ most affected by the disease, irritability of this viscus, with rejection of its contents, accompanies the progress, terminating in the black vomit. The intellectual functions in these cases are little disturbed. When the brain is the assailed organ, the stomach retains its tranquillity, and coma supervenes. The tinge of the skin, Dr. Musgrave says, is not constant in its occurrence, nor uniform in its intensity. It is commonly deeper in native or assimilated constitutions, approaching an orange; and paler in new-comers, resembling more the tint of a pale lemon. This, as a diagnostic mark betwixt the concentrated, continued, and remittent forms of the disease, as it has been attempted to establish, Dr. Musgrave says, is almost as futile as the black vomit.

"It does not always occur in either form; and, when it does, only serves to mark the intensity of the disease and its degree of danger. In a word, it is rather a means of prognosis than of diagnosis."

A marked difference of opinion subsists betwixt Dr. Musgrave and Mr. Pym on an important point of practice, and which, in the opinion of the latter gentleman, is subservient to the distinction betwixt yellow fever and the bilious remittent. This is on blood-letting; which, according to Mr. Pym, is of service in the latter affection, as he distinguishes them, and quite inadmissible in the former. Dr. Musgrave, denying the distinctive existence of two diseases, says, that in both forms they found blood-letting their sheet-anchor.

In proof of the identity of yellow fever with the intermittent fever from marsh miasmata, instances are given of the disease making its attack in situations peculiarly obnoxious to marsh effluvia, and remote from all source of contagion, in a regular intermittent type, lapsing into its continued form, and running to its fatal close with all the aggravated characteristics of the epidemic. It has been observed, too, breaking from its continued form into regular paroxysms of intermission. During the prevalence of this epidemic in Antigua, the black and coloured population was more than ordinarily infested, in situations which are notoriously the seat of such disease, with obstinate intermittents. Dr. Musgrave sums up his opinion on the nature of the epidemic in these words:

"In fine, so firmly have these, and other facts which I cannot at this moment distinctly call to mind, impressed me with the conviction that intermittent, remittent, and bulam or yellow fever, differ but in degree; and that they are only modifications of effect from the same cause, the modifications being influenced by the degree of concentration of the deleterious miasmata, and the varieties of assimilation, &c. in the subjects on which they operate; that it is a matter of the most serious surprise to me how any diversity of sentiment could ever have obtained upon the subject."

Some interesting remarks on particular symptoms, and on the general history of the disease, occur in this paper, which our limits preclude us from dwelling upon. The treatment which Dr. Musgrave's practice induced the chief dependence on, was a free detraction of blood in the very early stage of fever, abstaining cautiously from such depletion where the disease had existed for eight-and-forty hours; the bowels were cleared out with calomel and jalap, and kept regularly evacuated during the course of the complaint, but in this, too, caution is requisite, lest they be excited to an excessive and fatally debilitating catharsis. Cold ablutions, perseveringly applied, were found not so distressing to the patient, and equally impressive on the fever, as the cold affusion. Where a sense of chilliness contra-indicated the application of cold to the surface, tepid ablutions were productive of benefit. The circulating mass being diminished, and the intestinal canal cleared out, the cinchona, without waiting for a remission, was directly administered. By waiting till the stomach showed signs of irritability, the chance was thrown away of employing a highly valuable remedy.

When the cinchona in powder oppressed the stomach, an infusion of it in combination with serpentaria, to which a proportion of the sp. æther. nitros. was added, proved in general a form of admirable efficacy. Blisters to counteract local determination, and in the latter stages employed more extensively as stimulants, were useful adjuvants. Dr. M. says, that he could not avoid remarking that no individual died, in whom strangury supervened on their use; and he has since understood that the most beneficial power has been lately ascribed to them, depending on the absorption of the cantharides. Of the specific action of mercury on the disease, Dr. Musgrave professes to have little decided information. Though they had adopted in some of the later cases of the epidemic, on the commendation of some practitioners in the public service, they did not rely on it to the exclusion of those measures which they had found most generally efficacious. It appeared to have been advantageously used in some instances of the remittent form, where bleeding in the first instance had failed to subdue the urgent symptoms. Dr. M. says, he has satisfied himself of the less degree of irritation produced on both the stomach and bowels by the employment of large than of small doses of calomel.

*(To be concluded in our next.)*

*Pathological and Surgical Observations on Diseases of the Joints;* by B. C. Brodie, F.R.S. assistant Surgeon to St. George's Hospital, and Lecturer on the Theory and Practice of Surgery. 8vo. pp. 329. London, 1818.

When our readers have considered the nature of the office and duties of a critical reviewer, and the labours and anxious sensations with which the exertion of them is generally accompanied, they may form an adequate idea of the pleasure we experience on taking up the present work of Mr. Brodie: for we are not only relieved, on this occasion, from the more arduous part of our inquisitorial functions— that of passing sentence on the merits of an author; but from those also which must be deduced from historical research, and are intended to aid the reflections and facilitate the enquiries of the student respecting the subject under consideration. The opinion universally expressed on the value of Mr. Brodie's Remarks on Diseases of the Joints, published some years since, exempts us from the necessity of the former; and the originality of his Observations renders the latter of but little real utility. The space we can devote to this work will, therefore, be occupied by a mere analysis of what appears to be the most interesting of the subjects of which it treats.

Mr. Brodie divides diseases of the joints into the following species:—*Inflammation of the synovial membrane: ulceration of the synovial membrane: cases in which the synovial membrane has undergone a morbid change of structure: ulceration of the cartilages of the joints: and scrofulous disease of the joints, having its origin in the cancellous structure of the bones.* After some general anatomical and pathological remarks on the different structures which enter into the formation of the joints, the author proceeds to treat of inflammation of the synovial membrane, when it occurs as a primary affection.

"Although no period of life," he observes, "is altogether exempt from this disease, it does not occur equally in persons of all ages. It very seldom attacks young children; becomes less rare as they approach the age of puberty; and is very frequent in adult persons. This is the reverse of what happens with respect to some of the other diseases to which the joints are liable; and a knowledge of these circumstances will be found of some importance to the surgeon, in assisting him to form a ready diagnosis."

This disease, the author continues to remark, may take place as a symptom of a constitutional affection, from the use or abuse of mercury, &c. In other cases it is entirely local, and is sometimes apparently induced from a sprain, but more commonly from cold; and thence those joints but thinly covered by integuments, are most frequently thus affected. In occurs in different degrees of intensity, but generally in a slow or chronic form; sometimes continuing for weeks or months; and, with occasional recoveries and relapses, may harass the patient during several successive years.

The symptoms are pain in the joint, principally referred to one spot: this usually continues to increase during the first week or ten days, when it is at its height. In the course of one or two days after the commencement of pain, the joint may be observed to be swollen, which arises, in the early stage of the disease, entirely from the collection of a fluid in its cavity; and, in the more superficial joints, an undulation of the fluid may be felt. After the inflammation has existed for some time, the undulation is less distinct, in consequence of the synovial membrane having become thickened from effusion of coagulable lymph. As the swelling consists more of solid substance, the natural mobility of the joint is in a greater degree impaired. The form of the swelling is not that of the articulating ends of the bones: it arises chiefly from the distended state of the synovial membrane, and varies in different joints according as that membrane is more or less perfectly surrounded by ligaments and tendons.

After the inflammation has subsided, the fluid is absorbed, and in many instances the joint regains its natural figure and mobility; but, in the majority of cases, stiffness and swelling remain: the swelling sometimes has the same form which it possessed while the inflammation existed; at others, it has that of the articulating ends of the bones, or nearly that of the joint in a natural state. The former may, perhaps, arise from the inner surface of the synovial membrane having become lined with coagulated lymph; the latter from a general thickening of its structure.

The patient is liable to suffer relapses of the disease from the impression of cold, inordinate exercise, &c.; and sometimes a slight degree of inflammation lingering in the parts will extend to the other structures of the joint, and not unfrequently finally induce ulceration of the cartilage covering the surfaces of the bones. The latter species of disease may be distinguished from another affection, to be noticed hereafter, by the history of the malady.

When the inflammation is of the more acute kind, the swelling takes place immediately after, or at the same instant with, the first attack of pain: the pain is also very severe, particularly on the motion of the parts; and it is accompanied with some degree of symptomatic fever. Mr. Brodie continues to observe, that the boundaries of acute and chronic inflammation do not admit of being defined; but that those terms may designate the extremes of variations of form, which should be attended to in practice, as requiring somewhat different modes of treatment.

The above is a slight sketch from the author's account of the pathology and symptoms of inflammation of the synovial membrane: we shall now treat, in the same manner, of the curative measures adapted for that disease. When it appears to have arisen from a protracted or ill-conducted course of mercury, sarsaparilla may be given with some advantage. Opium conjoined with diaphoretics, the colchicum autumnale, &c. are advisable when it appears to be connected with rheumatism; and in some instances, when several joints are affected at the same time, much benefit is derived from moderate doses of some mercurial preparation. Topical remedies are, however, in all cases, of most importance.

In the more acute forms of the disease, leeches should be applied in the neighbourhood of the part; and, if there be much symptomatic fever, blood may be drawn from the arm. Saline purgatives and diaphoretics; and cold evaporating lotions, when there is not great tension of the parts, should also be employed.

Perfect quietude of the limb is necessary; and, in the chronic form of the inflammation, after having removed the more urgent symptoms by the abstraction of blood by cupping-glasses, and the use of cold lotions, vesicatories may be applied; which should be repeated in as rapid succession as the period required for the healing of the blister will permit: this mode is more effectual than maintaining an ulcerated surface by means of the savine cerate. The vesicatories have been found more beneficial when applied at a short distance from the joint; acting thus as counter-irritants, without producing immediate excitation of the inflamed parts. The same measures are most efficacious for the removal of the diseased state of the membrane consequent on the inflammation. When it is in a great measure relieved, a moderate degree of exercise of the joint is rather beneficial than otherwise. It will be useful, in this stage of the disease, to produce irritation of the skin covering the joint by stimulating liniments, or the ointment of tartarized antimony. The application generally directed by Mr. Brodie is composed of sulphuric acid, with four, five, or more, times its quantity of olive oil. Hospital patients, in general, whose skin is thick and somewhat callous, will bear it in the proportions of one part of the former to three of the latter. The effect of this liniment is to excite some degree of inflammation of the skin: the cuticle becomes of a brown colour, and separates in thick, broad scales. Issues and setons are of no service, except when it appears that a secondary disease has begun to take place, in the form of ulceration of the cartilages. When swelling and stiffness remain after inflammation has subsided, free exercise of the limb and friction over the joint may be employed with advantage.

Mr. Brodie relates several cases where inflammation of the synovial membranes of different joints appeared as a concatenation in the transition of diseases of the membranes from one part of the body to another; and which, in some degree, illustrate the beautiful doctrines of Bordeu respecting those structures.

One gentleman had eight attacks of inflammation of the synovial membranes.

"The first took place when he was under twenty years of age, the others at various intervals in the course of the ensuing seventeen years. In one of them, the first symptom was inflammation of the urethra, attended with a discharge of pus, which could not rationally be attributed to venereal infection. This was followed by purulent ophthalmia; and that, by inflammation of the synovial membranes. In three of the attacks, a purulent ophthalmia was the first symptom; which was followed by inflammation and discharge of matter from the urethra; and then the synovial membranes became affected. In the other four attacks, the affection of the synovial membranes took place without any preceding inflammation of the eye or urethra. The disease was not confined to the synovial membranes of the joints, but those of the bursæ mucosæ were inflamed also. In some of the attacks, the muscles of the abdomen were painful and tender, and subject to spasmodic contractions; and there was occasionally an impediment to breathing, which seemed to arise from a similar affection of the diaphragm. The acute form of the disease, in this case, lasted from six weeks to three months; but nearly a year elapsed before the use of the limbs was perfectly restored. The last attack began in July, 1817; and in the beginning of May, 1818, while he was still lame, he was seized with a very violent inflammation of the sclerotic coat and iris of one eye, which was subdued by very copious blood-letting and the exhibition of mercury."

Mr. Brodie has witnessed two instances of death ensuing from *ulceration of the synovial membrane*, occurring as a primary affection.

"A young lady, nine years of age, being at play, on the 1st of January, 1808, fell and wrenched her hip. She experienced so little uneasiness, that she walked out on that day as usual. In the evening she went to a dance; but while there was seized with rigor, was carried home, and put to bed. Next morning she was much indisposed, and complained of pain in the thigh and knee: on the following day she had pain in the hip, and was very feverish. These symptoms continued; she became delirious; and she died just a week from the time of the accident.

"On inspecting the body on the following day, the viscera of the thorax and abdomen were found in a perfectly healthy state. The hip-joint on the side of the injury contained about half an ounce of dark-coloured pus; and the synovial membrane, where it was reflected over the neck of the femur, was destroyed by ulceration for about the extent of a shilling.

"A middle-aged man, who had met with a contusion of one shoulder, was admitted into St George's Hospital in the winter of 1812. He complained of pain and tenderness of the shoulder, and a very slight degree of swelling was observable; but his principal disease was a fever, resembling typhus in its character, of which he died in a few days after his admission.

"On inspecting the body, about half an ounce of thin pus was found in the shoulder-joint. The synovial membrane bore marks of general inflammation, and in one spot, where it was reflected over the neck of the os brachii, it was destroyed by ulceration for about the extent of a sixpence."

These cases, the author remarks, form by no means solitary examples: every surgeon will be able to call to mind numerous other instances, which show that an impression made upon a small part of the nervous system may derange, and ultimately destroy, the functions of the whole animal economy.

Numerous instances have occurred to his observation, where the synovial membrane had undergone a *peculiar morbid change of structure.* In these cases, he observes, the morbid action evidently originates in the synovial membrane, which loses its natural organization, and becomes converted into a thick pulpy substance, of a light-brown, and sometimes of a reddish-brown, colour, intersected by white membranous lines. As the disease advances, it involves all the parts of which the joint is composed, producing ulceration of the cartilages, caries of the bones, wasting of the ligaments, and abscesses in different places.

"This disease," Mr. Brodie remarks, "is peculiar to the synovial membranes; at least, I have never met with it in any other part of the body: but it belongs to the same order with tubercles of the lungs, schirrus of the breast, the medullary sarcoma or fungus hæmatodes of the testicle, and numerous other diseases, in which the natural structure of the affected organ is destroyed, and a new and different structure is added in its place. To these it also bears a near resemblance in its progress. Thus, tubercles of the lungs, in the first instance, occupy the vesicular and interlobular substance; but ultimately they inflame and ulcerate, abscesses form in them, and then the pleura, bronchiæ, and other contiguous parts, become affected.

"In every case in which I have had it in my power to watch its progress, the complaint has been slow, and sometimes has remained in an indolent state during a very long period; but ultimately it has always terminated in the destruction of the joint."

It is rarely met with except in the knee. Mr. Brodie has never seen it in the hip or shoulder. It is probable, he remarks, that the influence of external cold may operate as one of the causes by which it is produced; and this explains why it occurs frequently in the knee, and seldom in the deep-seated articulations.

It generally affects persons who are not much past the age of puberty; and, for the most part, can be traced to no evident cause, though it sometimes appears to take place in consequence of repeated attacks of inflammation. The following is Mr. Brodie's account of the symptoms and progress of this malady:—

"In the origin of this disease, there is a slight degree of stiffness and tumefaction, without pain, and producing only the most trifling inconvenience. These symptoms gradually increase. In the greater number of cases, the joint at last scarcely admits of the smallest motion; but in a few cases it always retains a certain degree of mobility. The form of the swelling bears some resemblance to that in cases of inflammation of the synovial membrane, but it is less regular. The swelling is soft and elastic, and gives to the hand a sensation as if it contained fluid. If only one hand be employed in making the examination, the deception may be complete, and the most experienced surgeon may be led to suppose that there is fluid in the joint, when there is none: but if both hands are employed, one on each side, the absence of fluid is distinguished by the want of fluctuation.

"The patient experiences little or no pain, until abscesses begin to form and the cartilages ulcerate; and even then the pain is, in many instances, not so severe as where the ulceration of the cartilages occurs as a primary disease; and the abscesses heal more readily, and discharge a smaller quantity of pus, than in cases of this last description. At this period the patient becomes affected with hectic fever, loses his flesh, and gradually sinks, unless the limb be removed by an operation.

"The progress of this disease varies in different cases. In general, one or two years elapse before it reaches its most advanced stage; but sometimes the period is much longer, and occasionally it becomes indolent, so that it remains many months without any sensible alteration.

"The diagnosis of this disease is seldom difficult. The gradual progress of the enlargement, and stiffness of the joint without pain, and the soft elastic swelling without fluctuation, in the majority of cases, enable us to distinguish it readily from all the other morbid affections to which the joints are liable."

This is equally incurable with the other instances of morbid change of structure to which the author has alluded. All the various measures he has tried, or seen other surgeons employ, have only led him to form the above conclusion. The progress of the disease may be somewhat checked by means of rest and cold lotions; and, when there is considerable pain in consequence of the cartilages having begun to ulcerate, some benefit has been derived from warm fomentations and poultices; but no method with which he is acquainted is capable of doing more than checking the progress, and somewhat relieving the symptoms, of the complaint. In every instance where he has had an opportunity to witness its termination, ulceration of the cartilages, the formation of abscesses in the cavity of the joint, and the consequent disturbance of the patient's health, have rendered amputation of the limb the only apparent mean of preserving his life. At this stage of the disease the surgeon must urge the operation; but, at an earlier period, it may be a matter of choice to the patient, whether or not he will live with the incumbrance of the useless limb until its removal is indispensable.

The chapter on *ulceration of the cartilages of the joints* will not admit of regular analysis. There are so many circumstances attending this disease, the knowledge of which is of such high importance, that the observations of Mr. Brodie, although related with the utmost conciseness, occupy a considerable proportion of the space of this volume. Our account of it must, therefore, be very general, and such as will rather excite such of our readers, as may not have already studied it, to the perusal of the work itself.

"In the cases which have been related," says Mr. Brodie, "the ulceration of the cartilages of the diseased joints was a secondary affection, the consequence of a morbid action originating in the neighbouring soft parts. There are other cases, and those not of rare occurrence, in which the ulceration of the articular cartilages exists as a primary disease.

"When the ulceration of the cartilage occurs in the superficial joints, it constitutes one of the diseases which have been known by the name of white-swelling. From those which I have met with, I am led to conclude that, when it takes place in the hip, it is this disease which has been variously designated by writers—the 'morbus coxarius,' the 'disease of the hip,' the 'scrofulous hip,' the 'scrofulous caries of the hip-joint.' At least, it is to this disease that these names have been principally applied; though, probably, other morbid affections have been occasionally confounded with it."

The following is a sketch of the different stages and general progress of this disease:—1. Ulceration takes place in the cartilages; generally that of the acetabulum first, and in that of the head of the femur afterwards: sometimes it begins in both at the same time.—2. The ulceration extends to the bones, which become carious; the head of the femur is diminished in size, and the acetabulum is rendered deeper and wider.—3. Abscess forms in the joint, which after some time makes its way, by ulceration, through the synovial membrane and capsular ligament, into the thigh, nates, or even through the bottom of the acetabulum into the pelvis. —4. In consequence of the abscess, the synovial membrane and capsular ligament become inflamed and thickened: the muscles are altered in structure; sinuses are formed in various parts; and at last all the soft parts are blended together into one confused mass, resembling the parietes of an ordinary abscess.

"In some cases," continues the author, "the ulceration of the cartilage begins on that surface which is connected with the bone; and, from having observed this circumstance, I was led at first to adopt an opinion, which I have heard stated to have been that of Mr. Hunter, and which appeared to be warranted by the small degree of vascularity which cartilage possesses,—that ulceration of it takes place, not from the action of its own vessels, but in consequence of its being acted on by the vessels of the bone to which it is connected. I afterwards found that, in many instances, previously to ulceration, the cartilage undergoes a remarkable change of texture, becoming soft, and assuming a fibrous appearance: I was thence led to conclude that this opinion is not altogether correct, and I since witnessed two cases which appeared to show that cartilage, as well as other parts, is capable of ulcerating from the action of its own vessels."

Ulceration of the articular cartilages occurs at any period of life, but principally in children or in adults under the middle age. It is generally confined to a single joint, most frequently that of the hip. Sometimes the patient traces its origin to a local injury; but, for the most part, no cause can be assigned for the disease, and often that to which it is attributed is more imaginary than real.

When the hip is affected, the only symptoms in the early stages are—trifling pain, and a slight degree of lameness in the lower limb. The pain a good deal resembles rheumatism, having no certain seat; being referred to different parts of the limb in different individuals, and even in the same person at different periods. As the pain increases in intensity, it is more confined in its situation. In the greater number of instances it is referred to the hip and the knee also, and the pain of the knee is generally the most severe of the two. The pain is much aggravated by motion of the joint, particularly by whatever occasions pressure of the ulcerated cartilaginous surfaces against each other. Soon after the commencement of pain, the parts about the hip-joint become tender. The absorbent glands in the groin sometimes become enlarged. The pain about the knee, although originating from sympathy, is frequently at length accompanied with swelling and inflammation.

When the disease has existed for some time, the nates undergo a remarkable alteration in their form: they become wasted and less prominent; so that, instead of their usual convexity, they have a flattened appearance, and seem wider than those of the other side; and, in some few cases, in the advanced stage of the disease, they are really wider, in consequence of the acetabulum being filled with coagulated lymph and matter, and the head of the femur being pushed out of its natural situation. Another symptom which occurs in this disease is an alteration in the length of the limb. 1. In the early stage of the complaint, the patient often imagines the limb of the affected side to be longer than the other: but this is only apparent, and is produced by the position of the pelvis being altered in such a way that the crista of the one ilium is depressed below the level of that of the other; which arises from the posture in which the patient places himself when he stands erect. He supports the weight of his body on the sound limb, the hip and knee of which are, in consequence, maintained in a state of extension; at the same time that the opposite limb is inclined forward, and the foot placed on the ground considerably anterior to the other; not for the purpose of supporting the superincumbent weight, but to keep the person steady and preserve its equilibrium: this cannot be done without the pelvis being depressed on the same side.—2. In a few cases, where the patient is in the erect position, it may be observed that the foot of the affected limb is not inclined more forward than the other, but that the toes only are in contact with the ground, and the heel raised, at the same time that the knee is a little bent. This answers the same purpose, that of enabling the patient to throw the weight of his body on the other foot; but it produces an inclination of the pelvis in the opposite direction to that before described. The crista of the ilium is higher on the affected than on the sound side in these instances, and there is an apparent shortening of the diseased limb.—3. In the most advanced stage of the disease, when the head of the femur has been destroyed by ulceration, the muscles draw the bone upwards, and there is real shortening of the limb: the foot may here be rotated inwards, but, if left to itself, it is generally turned outwards.—4. In other cases the limb is shortened; the thigh is bent forwards; the toes are turned inwards, and do not admit of being turned outwards; and there is every symptom of dislocation of the hip upwards and outwards, which really takes place in consequence of the cavity of the acetabulum having become filled with coagulated lymph and matter, and the round ligament destroyed by ulceration.

The shortening of the limb, which takes place in the advanced stage of the disease, is usually, but not always, the precursor of abscess. The formation of matter is also indicated by an aggravation of pain, by greater wasting of the limb, and the thigh becoming more bent forward, and being incapable of extension without causing a great and almost intolerable increase of the sufferings of the patient.

After some observations on the varieties in the character of the disease when it occurs in other joints, Mr. Brodie enters on the consideration of the mode of treatment. The keeping the limb in a state of perfect quietude is one of the first and most important circumstances to be attended to. It is in this malady that caustic issues, setons, and blisters kept open with savine cerate, have been productive of so much benefit.

When the cartilages of the hip-joint are affected, the patient should, in the first instance, be confined to a couch, if not to his bed; and, if the disease be far advanced, the limb should be supported by pillows, so as to favour the production of anchylosis, by allowing it to vary as little as possible from one position. In young children, blisters are capable of affording complete relief: they may be applied on the nates, round the great trochanter, and in the groin; and kept open by savine cerate. In children above the age of eight or ten years, and in adults, the same treatment is useful in the very early stage of the disease; but, in the more advanced periods, issues made with caustic are more efficacious. The hollow behind the great trochanter of the femur is, in many respects, the most convenient situation for the application of the caustic; but, in some cases, the application of it on the outside of the hip is attended with better effects; perhaps from the skin in this situation being nearer to the joint than that of the former. An ulcer may be made with pure potash, half an inch in breadth and two inches in length, in the adult, behind the great trochanter; and, if this fail to give relief, a second, of a smaller size, may be produced in the situation of the anterior edge of the tensor vaginæ femoris muscle. The good derived from the issue does not seem to be in proportion to the quantity of pus discharged from its surface. From the observation having been frequently made, that more abatement of the symptoms is produced during the first few days from the application of the caustic than in several weeks afterwards, Mr. Brodie was led to consider that keeping the issue open, by rubbing its surface with pure potash, or sulphate of copper, two or three times a-week, would be more beneficial than the use of beans for this purpose. An extensive trial of both methods has appeared to demonstrate the superior efficacy of the former to that of the latter method. The pain produced by the use of the caustic is very considerable; but the subsequent relief of the symptoms is such, that patients have frequently been found to make the application themselves.

These measures, in general, produce an immediate alleviation of the symptoms; and, (provided that suppuration has not taken place,) if the patient continues in a state of quietude, the pain in general entirely subsides after the lapse of a few weeks. When the pain is exceedingly severe, and there is reason to believe that the ulcerated surfaces are in a state of much inflammation in consequence of the joint having been exercised, bleeding and the warm-bath may be had recourse to. A vesicatory may also be applied to the groin. The production of a blister on the knee or thigh will often produce considerable, and sometimes entire, relief of the pain which is referred to those parts from sympathy with the affection of the hip. The production of counter-irritation in the groin has been found particularly beneficial; but there are many objections to the application of caustic, which do not apply to the introduction of a seton in this situation.

"I was led to adopt this mode of treatment some years ago," says Mr. Brodie, "partly from observing that the skin of the groin is nearer to the hip-joint than the skin elsewhere; partly from an expectation (though not a very confident one) that the making a seton over the trunk of the anterior crural nerve might be particularly calculated to relieve the pain referred to those parts to which the branches of that nerve are distributed. The results of this practice more than realized whatever hopes I had entertained of its success. In many cases the seton occasioned very speedily a complete relief of the pain: in other cases, indeed, it failed to produce the like good effects; but these cases have borne only a small proportion to those in which it has succeeded. On the whole, I am led to conclude that, where the pain is very severe, the seton in the groin is more calculated to afford immediate relief than the caustic issue; but that it is not equally efficacious in checking the progress of the disease as in lessening the violence of its symptoms; and that the caustic issue can be better depended on for the production of a cure."

After some observations on the modification of the above plan of treatment that may be advisable when the disease affects other joints, Mr. Brodie proceeds to the consideration of the treatment of the abscess which ensues from it in its latter stages. He first adduces some remarks on the various measures that have been proposed for the treatment of abscesses in general, and how far they are applicable to that which forms the subject of the present observations. Evacuating the matter by means of a valvular opening, as recommended by Mr. Abernethy for lumbar abscess,—emetics,

—electricity,—and pressure, have not been found to produce the good effects on this as on other occasions. Early puncture of the abscess is certainly not advisable; the opening of it should, if possible, be avoided until the symptoms have been somewhat alleviated by the means above directed. When it is considered proper to evacuate the matter,

"—an opening should be made with an abscess lancet, and the limb wrapped in flannel wrung out of hot water, which may be continued as long as the matter continues to flow of itself. In general, when a certain quantity has escaped, the discharge ceases; the orifice heals, and the puncture may then be repeated some time afterwards; but, where the puncture has not become closed, I have seldom found any ill consequences to arise from it remaining open.

"When the ulceration of the cartilages has made very considerable progress," continues Mr. Brodie, "if the patient recovers so as to preserve the limb, he seldom has the use of the joint afterwards, the bones composing it being united by anchylosis; but, if it has been checked in a less advanced stage, even though there is reason to believe that the cartilages have been extensively destroyed, the patient may retain the natural motion of the joint. I have not hitherto examined any cases in which it appeared that there had been any attempt at the regeneration of the absorbed cartilages; and I have occasionally been able both to feel and to hear the hard surfaces of the bones grating against each other in such a manner that it was evident they had no cartilaginous coverings. In some instances a compact layer of bone is formed on the carious surface, nearly similar to what is seen on the healthy bone after the cartilage has been destroyed by maceration. I have many times, in dissection, observed a portion of the cartilage of a joint wanting, and in its place a thin layer of hard semi-transparent substance, of a grey colour, and presenting an irregular granulated surface. It is probable that, in these cases, the original disease had been ulceration of the cartilages. In a subject in the dissecting-room, I found no remains of cartilage on the bones of one hip, but in its place a crust of bony matter was formed, of a compact texture, of a white colour, smooth, and having an appearance not very unlike that of marble."

Mr. Brodie next treats of scrofulous disease of the joints; some other morbid affections of those organs, as tumors, portions of loose cartilage, and gouty concretions in their cavities; caries of the spine; and inflammation of the bursæ mucosæ: but the space which the analysis of the more important and original parts of this treatise has already occupied, prevents the extension of it to the subjects above enumerated. Indeed, we consider that it would be useless to adduce further extracts from a work that will constitute part of our classical medical literature.

